# Incomplete immunization uptake and associated factors among children aged 12–23 months in sub-Saharan African countries; multilevel analysis evidenced from latest demography and health survey data, 2023

**DOI:** 10.1186/s13052-024-01642-9

**Published:** 2024-05-12

**Authors:** Tigist Kifle Tsegaw, Helen Birhan Alemaw, Yordanos Bitweded Wale, Solomon Gedlu Nigatu, Tilahun Yemanu Birhan, Asefa Adimasu Taddese

**Affiliations:** Department of Epidemiology and Biostatistics, Institute of Public Health, College of Medicine and Health Sciences, University of Gondar, Gondar, Ethiopia

**Keywords:** Incomplete immunization, Partial, Sub-Saharan Africa, And pooled prevalence

## Abstract

**Background:**

In 1974, the World Health Organization (WHO) established the Expanded Program on Immunization to control vaccine-preventable diseases, saving millions of lives annually. However, the coverage of basic vaccines recommended by the WHO in Africa was only 75%, which fell short of the goal of 90% by 2015. To formulate effective policies and implementation programs to reduce incomplete vaccination rates, it is important to conduct a study to determine the factors contributing to incomplete immunization among children aged 12–23 months.

**Methods:**

The study was conducted in 16 sub-Saharan African countries, using data extracted from the latest DHS data. It was a community-based cross-sectional survey that used two-stage stratified probability sampling sample designs. The vaccination coverage was assessed using vaccination cards and mother recalls. Multilevel multivariable logistic regression was used to determine the extent of incomplete immunization and the individual and community-level factors associated with partial immunization among children aged 12–23 months. Variables with a *p*-value less than 0.05 were considered statistically significant predictors of incomplete immunization.

**Result:**

A total of 35, 193 weighted samples were used to determine the pooled prevalence of partial immunization. The pooled prevalence of incomplete immunization was 36.06%. In the final model factors significantly associated were: being uneducated mother(AOR:1.75;95%CI:1.48,2.05), being an unemployed mother (AOR:1.16;95%CI:1.09,1.23), no history of family planning utilization (AOR: 1.71; 95% CI: 1.61, 1.84), non-antenatal care (AOR: 1.79; 95% CI: 1.58, 2.04), non-postnatal care (AOR: 1.25; 95%CI: 1.17, 1.35), rural residence(AOR:1.50;95%CI:1.37,1.63), home delivery (AOR: 2.04; 95%CI:1.89, 2.21), having children more than five (AOR: 1.56; 95%CI: 1.13, 2.17), and non-utilization of health insurance (AOR: 1.74; 95%CI: 1.48, 2.05).

**Conclusion:**

The pooled prevalence of incomplete immunization was found to be high in this investigation. Based on the findings of the study we recommended that policymakers and stakeholders prioritize enhancing prenatal and postnatal care, contraception, and reducing home birth rates to minimize the rate of incomplete immunization.

## Background

The vaccination of children has led to a significant reduction in morbidity and mortality from different diseases, thereby lowering the infant mortality rate [1]. In 1974, the World Health Organization (WHO) established the Expanded Program on Immunization to control vaccine-preventable diseases, saving millions of lives each year [2]. WHO reported that about 22.6 million children under the age of one worldwide did not receive Diphtheria- Pertussis-Tetanus Vaccine Three (DTP3) vaccine and more than 70% of these children lived in ten countries of the Democratic Republic of Congo, Ethiopia, India, Indonesia, Iraq, Nigeria, Pakistan, Philippines, Uganda and South Africa [4]. Despite its widespread use, low vaccination rates have been recorded in developing nations. Failures or delays in the vaccination of children in high-risk groups can limit the impact of vaccine programs on the burden of disease [5]. In Sub-Saharan Africa, despite the availability of vaccines and the efforts of governments and their partners’ the mortality rate of children under the age of five years remains the highest [6]. Children in Sub-Saharan Africa are more than 15 times more likely than children in high-income countries to die before the age of five from vaccine-preventable diseases [7]. The situation is even worse in developing countries where 20–35% of under-five mortality is a result of vaccine-preventable diseases [8]. In Nigeria including children who had no ever taken vaccine around 76.3% of children aged 12–23 were incompletely immunized [9]. In 2013, most of the World Health Organization’s (WHO) regions reached more than 80% of their target populations with three doses of the diphtheria, pertussis, and tetanus (DTP) vaccine but coverage with such vaccine remained well short of the 2015 goal of 90%, particularly in the African (75%) [3]. With the high under-five mortality in SSA full childhood immunization can mitigate morbidity and mortality through prevention of a vaccine-preventable infection. The third dose of vaccine against diphtheria, tetanus, and pertussis (DTP3) has not been administered to 19.7 million children worldwide in their first year of life in 2019 [9]. Of all the children who did not complete the three-dose DTP series, 6.2 million (31%) started but did not complete the DTP series [5]. Despite widespread use made in the preceding ten years, low vaccination rates have been recorded in developing countries, particularly in SSA [3]. There is a study that assessed incomplete immunization uptake and associated factors among children aged 12–23 months in different countries of SSA. However, the pooled prevalence of incomplete immunization uptake in SSA countries has not been studied yet. Therefore this study aims to determine the pooled prevalence of incomplete immunization among children aged 12–23 months in SSA.

## Methods

### Data source and sampling procedure

The data for this study were obtained from the latest DHS data of 16 sub-Saharan African countries from 2015 to 2020 (Angola, Benin, Burundi, Cameron, Ethiopia, Guiney, mail, Malawi, Mauritania, Nigeria, Uganda, Sierra Leone, South Africa, Tanzania, Zambia, and Zimbabwe). The data sets were downloaded in STATA format from the DHS website (http://www.dhsprogram.com). Countries were selected based on the availability of recent standard DHS data. The DHS data is nationally representative data that uses four main standard model questionnaires (the Household Questionnaire, the Woman’s Questionnaire, and the Man’s Questionnaire) to collect data that are comparable across countries. The questionnaire covers basic health indicators such as marriage, fertility, mortality, family planning, reproductive health, child health, nutrition, and HIV/AIDS. It had men, women, kids, and household dataset records. For this study, we used a kids’ recode file.

It uses a two-stage stratified sampling technique samples were stratified by geographic region and by urban/rural areas within each region. In the first stage primary sampling unit, clusters were selected from the enumerations area. The second stage was a complete listing and selecting of a total of 25–30 households from each cluster by equal probability systematic sampling, then the data was collected from each selected household. For our study, we use a total of 35,087 children from selected SSA countries to determine the magnitude and associated factor of incomplete immunization among children aged 12–23 months.

### Study design, period, and area

**Fig. 1:**
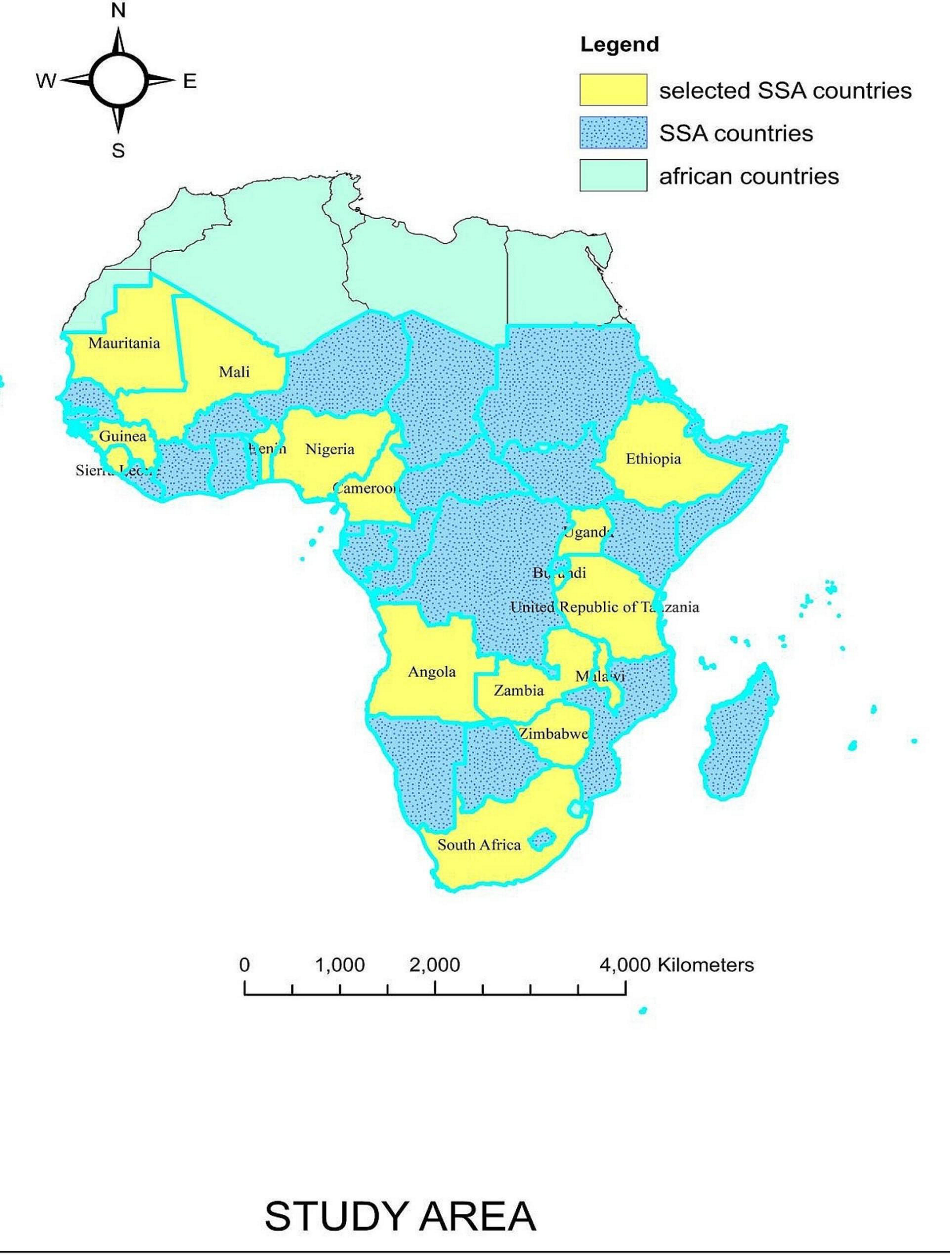
Study area of our study

The study was a community-based cross-sectional survey which was conducted in 16 selected sub-Saharan African countries from 2015–2020(Fig. 1).

### Study population, inclusion, and exclusion criteria

**Fig. 2:**
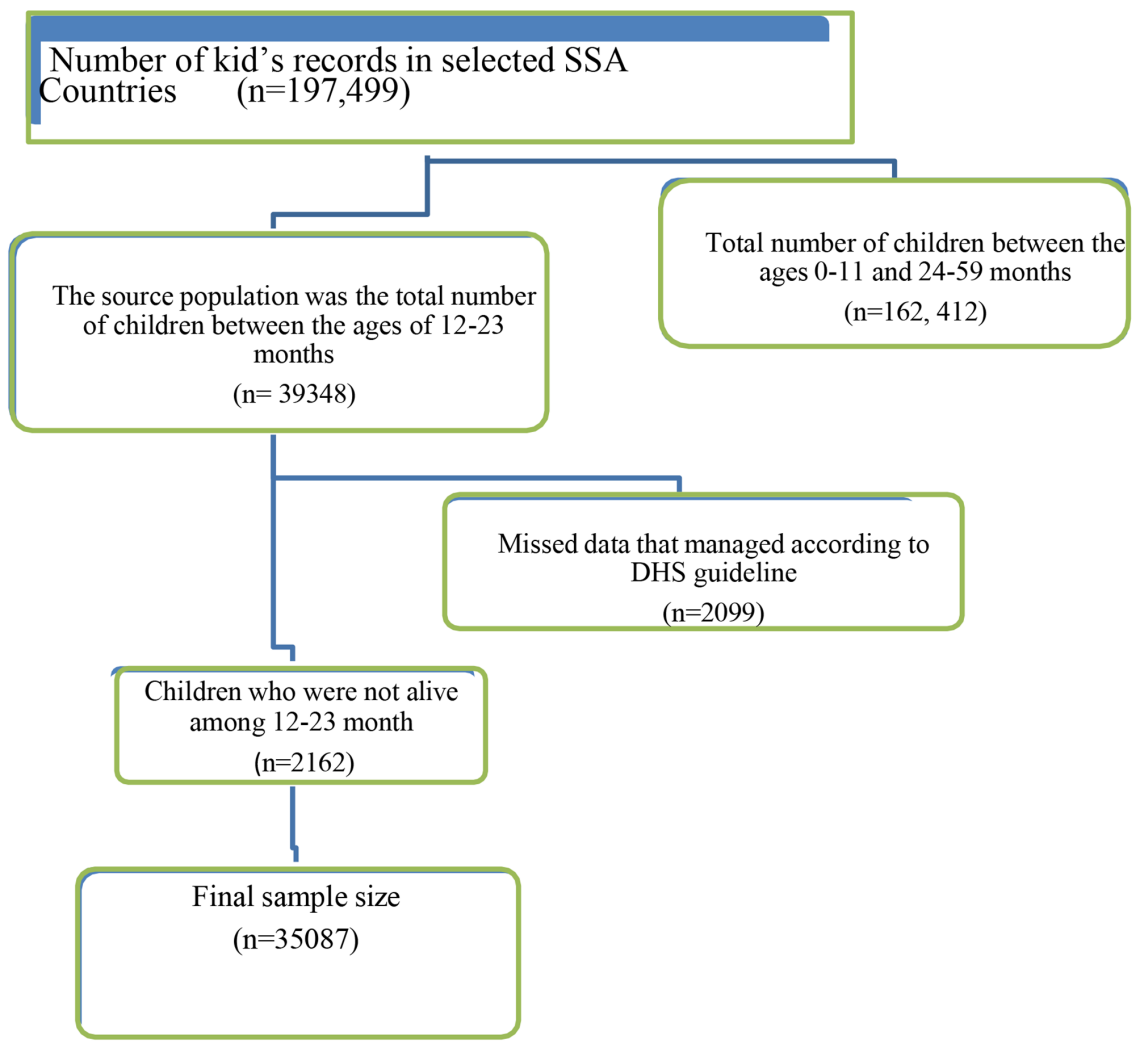
Flow chart for selecting of sample from kids record dataset

The Source population was all alive children aged 12–23 months in sub-Saharan African countries and the study population was all alive children aged 12–23 months in sub-Saharan African countries in selected enumeration areas (Fig. 2). A total of 35,087 children weighted samples were pooled from sixteen sub-Saharan African countries (Table 1) to determine incomplete immunization coverage and associated factors among children aged 12–23 months in sub-Saharan African countries. A child who didn’t possess a vaccination card and a mother who didn’t know the vaccination status of her child was excluded from the study.

**Table 1:** Sample size from each selected SSA country

Country name	Year	Unweighted sample	Weighted sample	Weighted proportion
Angola	2015/16	2630	2409	6.84%
Benin	2017/18	2402	2402	6.82%
Burundi	2016/2017	2473	2564	7.28%
Cameron	2018	1717	1785	5.07%
Ethiopia	2016	1831	1904	5.41%
Guinea	2018	1374	1323	3.76%
Mali	2018	1846	1953	5.55%
Malawi	2015/16	3152	3131	8.9%
Mauritania	2019/20	2017	2018	5.73%
Nigeria	2018	5787	5866	16.67%
Sera Leon	2019	1805	1779	5.06%
South Africa	2016	322	347	0.99%
Tanzania	2015/16	2050	2037	5.79%%
Uganda	2018/19	2714	2651	7.53%
Zambia	2018	1867	1835	5.22%
Zimbabwe	2015	1127	1185	3.37%
Total		35,087	35,193	

### Variable of the study

#### Outcome variable

The dependent variable was incomplete immunization coverage among children aged 12–23 months. According to WHO guidelines, children are fully immunized when they receive one dose of Bacillus Chalmette Guerin (BCG), three doses of DPT, three doses of polio vaccines, and one dose of measles-containing vaccination by the age of 9–12 months. We recorded each vaccine as “yes” and “no” for those who received and did not receive respectively. Then we added them up, recode them as vaccine status, and categorized them as “full” for those who received all, “partial “for those who missed at least one dose, and “no” for those children who had never taken a vaccine by the age of 12–23 month.

#### Independent variable

Socio-demographic characteristics (age, marital status, educational level and employment status of the mother, wealth of the family), and obstetric-related factors (ANC, place of delivery, parity, and use of family planning) were individual-level independent variables. At the community level place of residence and media exposure were considered. We generated a variable media exposure by summing up TV, radio, and newspaper we recorded each of them as “yes” and “no” for those who had been exposed and who hadn’t been exposed respectively. Then we added up and categorized them as “yes” if they were exposed to at least one of the three and “no” for those who had no exposure to at least one media.

### Data management and analysis

#### Data analysis

The data were kept, cleaned, recorded, and appended by STATA version 14.2 and exported to R version 4.3.0 for analysis and descriptive statistics (percent, proportion, graph, and frequency table). The sample was weighted by sampling weight v005 to make valid inferences. As the data set had a hierarchical nature, we used two-level mixed-effect multinomial logistic regression models. We fitted a total of 4 models the first is a null model without any explanatory variable, the second is for individual level explanatory variable third community level explanatory variable and finally, both individual and community level explanatory variables were fitted. We made a model comparison by using the log-likelihood ratio (LR) as the model was nested with a different number of parameters. The final model was the best-fitted model with a low likelihood ratio (LR). We calculated the intra-class correlation coefficient (ICC) to verify the significance of performing mixed effect analysis instead of simple multinomial logistic regression. Proportional change in variance was also calculated to know the variability explained by random effect in the final model. Variables with *p*-value < 0.2 in bi-variable analysis in the final model were included for multivariable analysis. In multivariable analysis, a *p*-value less than 0.05 was considered a statistically significant associated factor for partial immunization.

### Ethical consideration

This study was based on the existing survey data collected by the Demographic and Health Surveys (MEASURE DHS) project (www.measuredhs.com). All study participants gave written informed consent before participation and all information was collected confidentially. We requested the DHS program to use the data. The raw survey data and written consent of MEASURE DHS were obtained with authentication letter number 184,828 on May 23, 2023.

## Result

### Socio-demographic characteristics

A total of 35,193 kids aged 12–34 months weighted samples were selected from 16 sub-Saharan African countries for analysis. Among them 50.74% were male and one-third of the participants (68.8%) were live in rural areas. The mean age of the participants was 17.1 months. 59.57% of the participant’s mother age was between 15 and 29 years. More than half of the children (62.01%) had vaccination cards and were seen by the investigator whereas 13.59% of the children didn’t have vaccination cards and the remaining had a vaccination card but were not seen by the data collectors. The majority of the mothers (73.07%) were married 64.5% were employed and 36.35% of the mothers had no education (Table 2).

### Immunization uptake across the countries

**Fig. 3:**
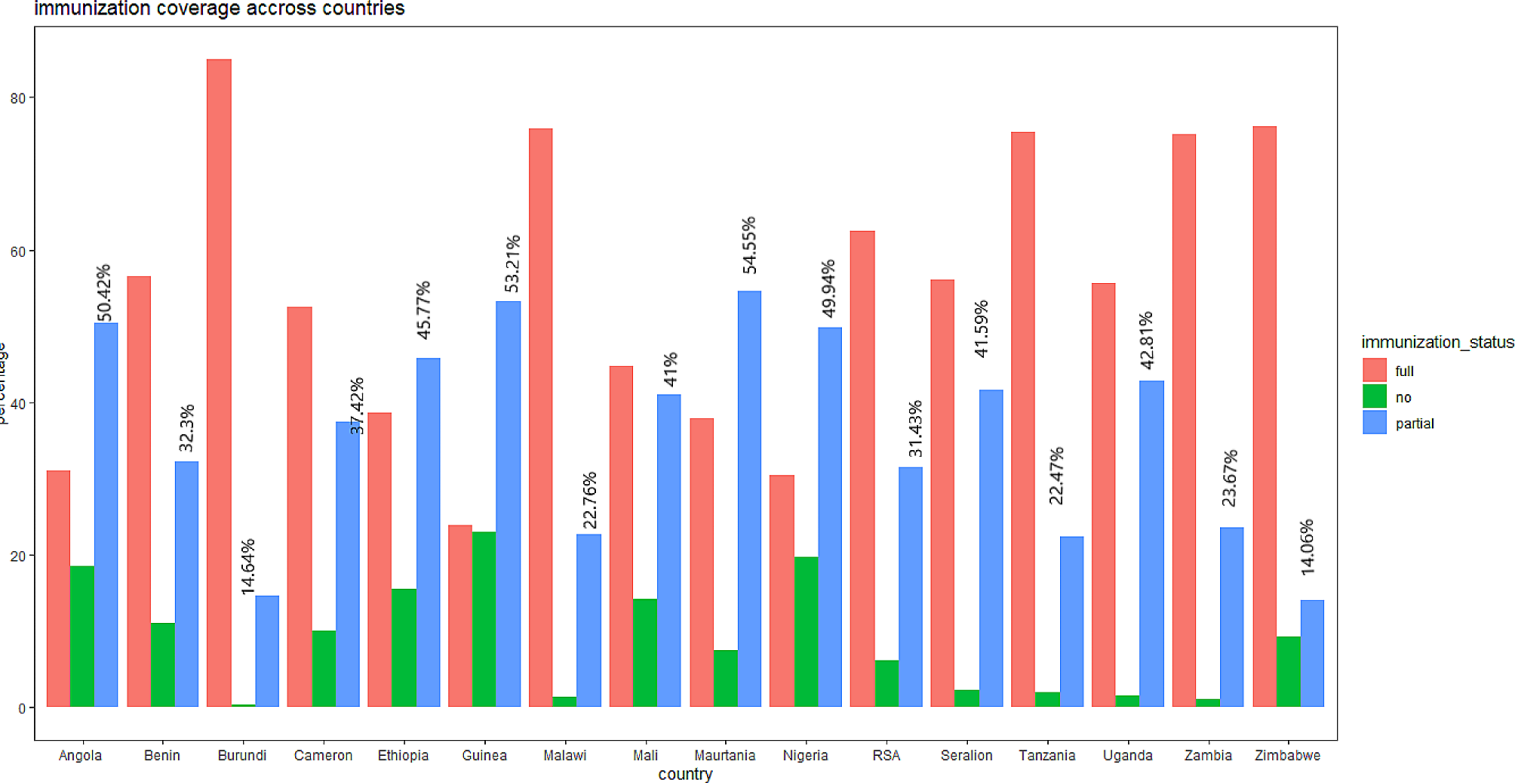
Percentage of immunization uptake across SSA countries Evidence from DHS data

From the findings of this study, the no immunization rate ranged from 22.94% in Guinea to 0.34% in Burundi. High inter-country variations of incomplete immunization uptake were also detected in SSA countries ranging from 14.06% in Burundi to 54.55% in Mauritania. Burundi was a country with a high percentage of full immunization coverage among the other countries while Nigeria was countries with the lowest percentage of full immunization coverage (Fig. 3).

### Percentage of each vaccine uptake

**Fig. 4:**
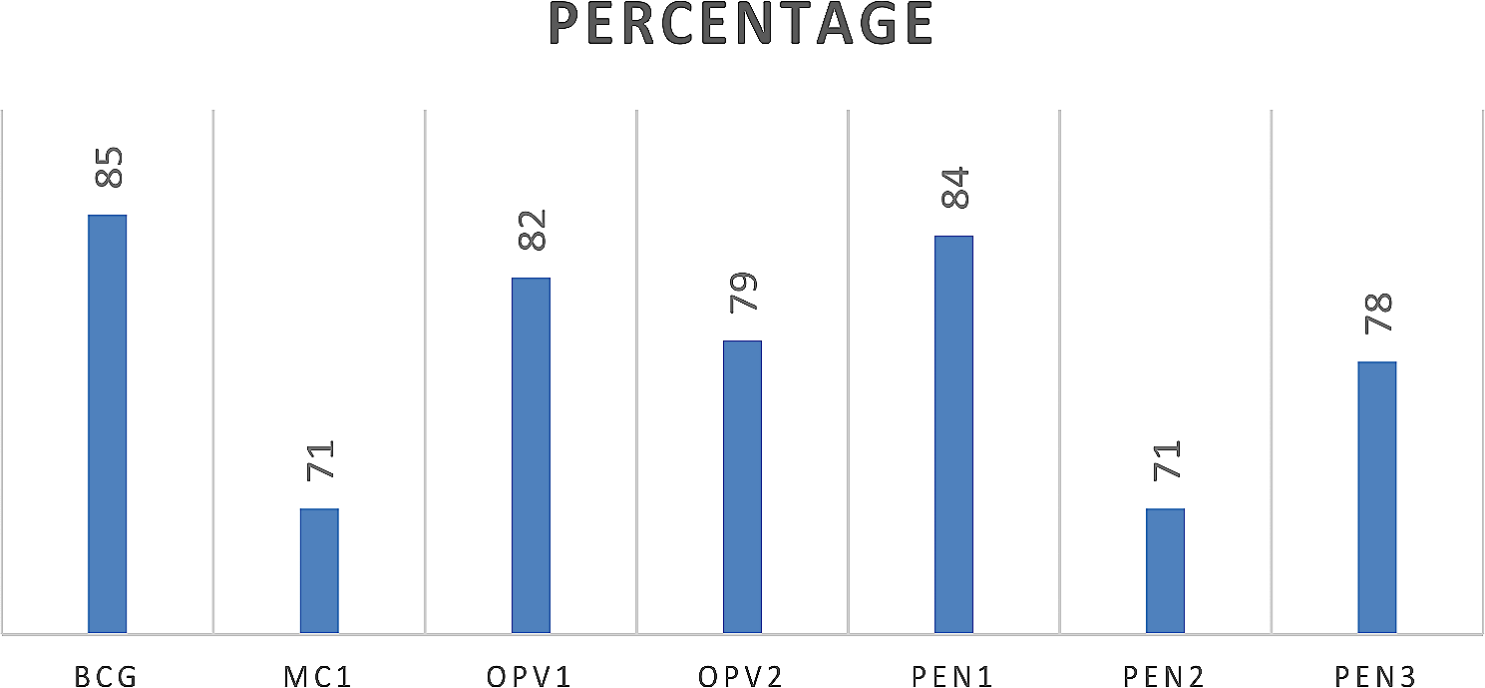
Percentage of each basic vaccine uptake in sub-Saharan Africa

The most frequently taken vaccine was BCG (85.3%) and pentavalent one (83.48%) whereas measles-containing dose (71.4%) and oral polio vaccine dose three were the least taken vaccine among the basic vaccine dose (Fig. 4).

### Pooled incomplete immunization coverage

**Fig. 5:**
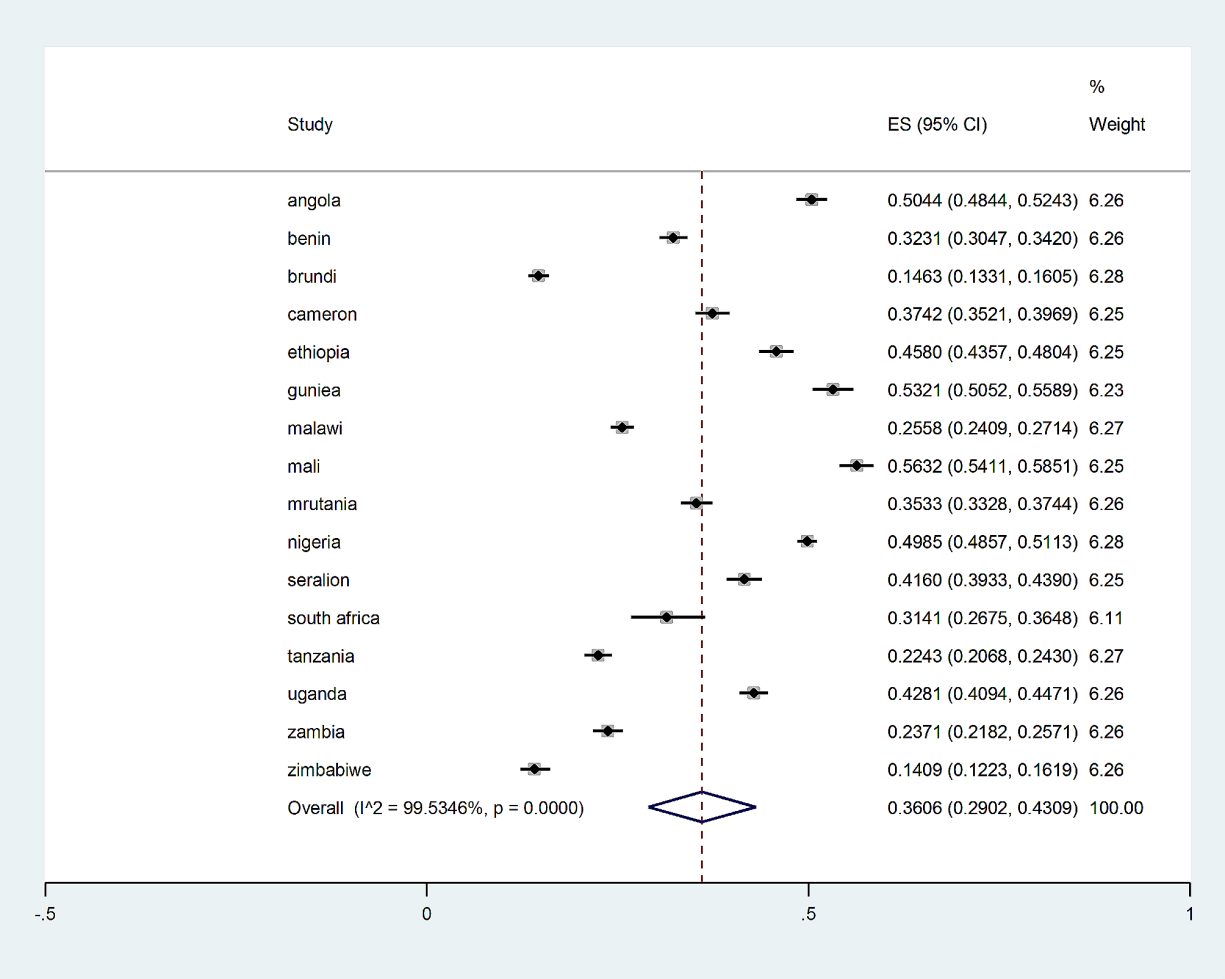
Forest plot of incomplete immunization among children aged 12–23 months in SSA countries

The prevalence of pooled incomplete immunization uptake in Sub-Saharan African countries was 30% with a 95% confidence interval of 29 to 43% from vaccination cards and women’s reports were 9% with 95% CI (6–11%) among children who had never gotten any one of the recommended basic vaccine doses (Fig. 5).

### Incomplete immunization uptake along explanatory variables

From the findings of this study among incompletely immunized children, 50.63% were male and two-thirds of them were (66.66%) from rural areas. And more than two-thirds (72.81%) were from married mothers. More than half of the mothers (59.86%) were between the ages of 15–29 and the majority (78.03%) of them had children less than 5. Of the total of partially immunized children around two-thirds (63.26%) and 40.5% of their mothers were unemployed and had no education respectively. Half of the mothers (50.91%) had at least one ANC follow-up. The majority of them (72.37%) had no PNC follow-up after delivery among the total of partially immunized children’s mothers 96.85% of partially immunized children’s families had no health insurance and 60.38% of the mothers had media exposure (Table 2).

**Table 2:** Incomplete immunization uptake along the explanatory variable among children aged 12–23 months

Variable	Weighted frequency	Weighted proportion	Incomplete immunization	
			Frequency	Percent
**Sex of child**				
Male	17856	50.73%	6680	50.63%
Female	17337	49.27%	6511	49.37%
**Marital status**				
Single	9478	26.93%	3586	27.19%
Married	25718	73.07%	9605	72.81%
**Age of the mother**				
15–29	20963	59.57%	7884	59.76%
30–39	11856	33.69%	4408	33.41%
>=40	2373	6.74%	899	6.81%
**Employment**				
Status				
Employed	22701	64.50%	8349	63.29%
Non employed	12493	35.50%	4842	36.71%
**Parity**				
4-Jan	28102	79.85%	10294	78.03%
9-May	6750	19.18%	2743	20.80%
>=10	351	0.99%	154	1.17%
**Wealth**				
Poor	15656	44.48%	6030	45.71%
Middle	7214	20.50%	2741	20.78%
Rich	12325	35.01%	4420	33.51%
**Educational level**				
No	12793	36.35%	5360	40.63%
Primary	12388	35.20%	4300	32.60%
secondary	8607	24.46%	3087	23.40%
Higher	1404	3.99%	445	3.37%
**No of ANC**				
No ANC	4801	13.64%	2019	15.31%
1–4	18518	52.61%	6716	50.91%
>=5	11874	33.74%	4455	33.77%
**Family planning**				
No	22983	65.31%	63	5.47%
Traditional	1148	3.26%	347	30.23%
Modern	11061	31.43%	739	64.29%
**Place of delivery**				
Home	10812	30.72%	4940	37.45%
Health facility	23869	67.82%	8101	61.41%
Other	513	1.46%	150	1.14%
**Postnatal care**				
No	24541	69.72%	9547	72.37%
Yes	10653	30.27%	3645	27.63%
**Sex of household**				
Male	28028	79.64%	10467	79.35%
Female	7165	20.36%	2723	20.65%
**Health insurance**				
No	33563	95.37%	12776	96.85%
Yes	1630	4.63%	415	3.14%
**Media**				
No	13706	38.95%	5226	39.62%
Yes	21487	61.05%	7965	60.38%
**Residence**				
Urban	10981	31.20%	4397	33.33%
Rural	24212	68.80%	8794	66.66%

### Factor associated with incomplete immunization uptake

#### Fixed effect model

AOR estimates were obtained from multilevel multinomial logistic regression to identify variables significantly associated with partial immunization uptake. A child who had an uneducated mother had 1.27 times (AOR; 1.27:95%CI; 1.07, 1.5) higher odds of being incompletely immunized rather than completely immunized and 1.85 (AOR; 1.85:95%CI; 1.14, 3.00) times higher odds being non-immunized rather than fully immunized as compared to that of a child who had a mother with higher education. This report revealed that a child from a mother who had no family planning usage had 1.72(AOR; 1.72:95%CI; 1.6, 1.84) higher odds of being incompletely immunized versus fully immunized and 2.85 times (AOR; 2.58:95%CI, 2.4, 3.36) non-immunized rather than fully immunized as compared to that of child from mother who used family planning method. Similarly, a child from an unemployed mother had 1.16 (AOR: 1.16:95%CI 1.58, 2.04) and 1.35 (AOR: 1.35; 95%CI; 1.20, 1.50) times higher odds of partially immunized and non-immunized versus fully immunized as compared to that of children from employed mother. According to our study a child from a family who didn’t have health insurance had 1.74 (AOR: 1.74; 95% CI; 1.48, 2.05) times higher odds of being incompletely immunized and 2.21(AOR; 2.21:95%CI; 1.45, 3.34) times higher odds of being non-immunized versus fully immunized as compared to counterpart. A child who was born at home had 2.04(AOR; 2.04:95%CI; 1.89, 2.02) times higher odds of being partially immunized and 4.47(AOR; 4.47:95%CI; 3.91, 5.12) times being none immunized rather than completely immunized as compared to that of children who born at a health facility. Moreover, a child whose mother didn’t attend ANC follow-up had 1.79(AOR; 1.79:95% CI; 1.56, 2.04) higher odds of being partially immunized versus completely immunized and5.67(AOR; 5.67:95&CI; 4.80, 6.71) higher odds of being unimmunized rather than fully immunized as compared to that of a child whose mother attend more than five ANC follow up. Additionally, the child whose mother didn’t attend PNC had1.26 (AOR: 1.26;95% CI:1.17, 1.35) higher odds of being partially immunized rather than fully immunized as compared to that of a child whose mother attended PNC. At a community level factor, a child from a rural area had 1.50 times (AOR: 1.50;95%CI:1.37,1.63) higher odds of being partially immunized rather than fully immunized as compared to a child from an urban area (Table 3).

**Table 3:** Mixed effect multinomial logistic regression for a factor associated with incomplete immunization among children aged 12–23 months in SSA countries

Variable	AOR(95%CI)	*P* value	AOR[95%CI]	*P* value
**INDIVIDUAL FACTORS**				
**Wealth**				
Poor	1.41(1.31,1.5)	0.000	1.001(0.92,1.07)	0.994
Middle	1.12(1.109,1.29)	0.000	0.99(0.92,1.08)	0.946
Rich	1.00		1.00	
**Education**				
No	2.27(1.94,2.67)	0.000	1.23(1.07,1.49)	0.006*
Primary	1.25(1.07,1.47)	0.006	0.95(0.80,1.12)	0.53
Secondary	1.27(1.08,1.5)	0.003	1.07(0.91,1.27)	0.39
Higher	1.00		1.00	
**Family planning**				
No	2.18(2.04,2.33)	0.000	1.72(1.61,1.84)	< 0.001**
Traditional	1.11(0.94,0.31)	0.223	1.06(0.89,1.26)	0.464
Modern	1.00		1.00	
**Employment**				
unemployed	1.16(1.09,1.24)	0.000	1.16(1.09,1.24)	< 0.001**
Employed	1.00		1.00	
**ANC**				
No	3.08(2.73,3.49)	0.000	1.80(1.58,2.04)	< 0.001**
1–4	0.98(0.92,1.03)	0.451	0.89(0.83,0.95)	< 0.001**
>=5	1.00			
**Place of Delivery**				
Home	2.68(2.49,2.89)	0.000	2.04(1.89,2.21)	< 0.001**
Facility	1.00		1.00	
Other	0.88(0.66,1.18)	0.402	0.85(,0.63,1.15)	0.282
**Postnatal care**				
No	1.44(1.35,1.54)	0.000	1.25(1.17,1.35)	< 0.001**
Yes	1.00			
**Health insurance**				
Yes	1.00		1.00	
No	2.08(1.77,2.45)	0.000	1.75(1.48,2.05)	< 0.001**
**Sex of child**				
Male	1.00			
Female	1.01(0.95,1.06)	0.995		
**Age of the mother**				
15–29	0.93(0.83,1.04)	0.203		
30–39	0.91(0.81,1.02)	0.123		
>=40	1.00			
**Parity**				
1–4	1.00		1.00	
5–9	1.32(1.22,1.44)	0.000	1.09(1.01,1.18)	0.017*
>=10	2.23(1.66,2.99)	0.000	1.56(1.12,2.17)	0.008*
**COMMUNITY LEVEL**				
**Media**				
Yes	1.00		1.00	< 0.001**
No	1.21(1.14,1.23)	0.000	0.96(0.89,1.02)	
**Residence**				
Rural	0.92(0.86,0.99)	0.035	1.00	
Urban	1.00		1.50(1.37,1.63)	< 0.001**

*p-value < 0.01, ** *p*-value < 0.001

### Random effect analysis and model comparison

The fixed effect analysis (A measure of association) for incomplete immunization uptake was presented in Table 3 whereas the random intercept analysis was presented in Table 4. The result of the empty model revealed that there was significant variability in the odds of receiving immunization uptake in Sub-Saharan Africa. As indicated in Table 4, the ICC in the null model revealed that 71% of the variability in incomplete immunization uptake was attributed due to difference between clusters/communities. While PCV in the final model revealed that about 37% of the variability of incomplete immunization uptake was explained by both individual and community level factors. Regarding model fitness, the model with the highest log-likelihood and the lowest AIC, Model 3, was found to be best fitted model (Table 4).

**Table 4:** The random effect model for factors associated with incomplete immunization uptake in sub-Saharan African countries

	null model	Model 1	Model 2	Model 3
Community level variance	0.71(0.57, 0.87)	0.44(0.35, 0.56)	0.68(0.55, 0.84)	0.45 (0.35, 0.57)
ICC (%)	0.18	0.12	0.17	0.12
PCV	-	38.03%	4.23%	36.62%
Log-likelihood	-32464.6	-28604.28	-32166.71	-26800.08
Deviance	64929.2	57208.56	64333.42	53600.16
BIC	64960.6	57574.85	64385.75	57587.38
AIC	64935.21	57278.56	56978.45	57274.15

## Discussion

The study evaluated the prevalence and contributing factors of incomplete immunization among children in Sub-Saharan Africa (SSA) between the ages of 12 and 23 months. The assessment of incomplete immunization uptake was based on mothers’ recall reports and vaccination cards; the vaccination card possession rate was 62.4%, which was lower than that of Togo in 2018 (77%) [10] and higher than that of Ethiopia in 2011 (41.8%) [11]. The pooled prevalence of partial vaccine uptake from mothers’ recollection reports and vaccination cards were 36.06% (95%CI 29.02%, 43.09%). The prevalence of incomplete immunization varied throughout the countries; it was 14.06% in Zimbabwe whereas it was 56.3% in Mauritania. The finding was in line with a study conducted in India (32%) [12], Togo (41.2%) [10], and Ethiopia (30%) [13]. The similarities may stem from the fact that both vaccines and poor access to healthcare are unavailable in low- and middle-income countries (LMICs) [14]. However, the results of this study exceeded the 10% WHO-recommended cutoff mark for vaccine incompletion rates [16]. Insufficient access to healthcare facilities and limited maternal vaccine awareness in Sub-Saharan African countries may be contributing factors to incomplete immunization [15]. In the final multilevel multivariable logistic regression model, nine covariates were found to be substantially correlated with partial immunization uptake. In this study, we found that non-utilization of health services such as ANC and PNC was a statistically significant determinant factor for partial immunization consistent with other studies conducted in Cameron, Nigeria, and Jamaica [16]. This is because women who did not receive PNC or ANC may have fewer interactions with medical professionals and may not receive the usual vaccination-related advice provided during ANC sessions. Maternal education was found to be substantially correlated with incomplete immunization in line with studies conducted in West Africa, Jamaica, and Kenya [17–19]; mother employment was also a major predictor of incomplete immunization. This conclusion is corroborated by a Bangladeshi study [20]. Hence an uneducated mother may not be as likely to seek medical attention or recognize the value of vaccinations [21]. Also, home birth raises the likelihood of receiving a partial vaccination, which is in line with research from West Africa [22]. Hence a mother gives birth at home, she may not receive the immunizations that are administered at birth and on time [16]. The use of family planning was also a strong predictor of incomplete immunization which is corroborated by Ghanaian research [23]. This indicated that through the integrated program of family planning and immunization services, the woman who visited the health facility for family planning services may be able to vaccinate her kid [24]. According to a study done on African-American and Latino children in inner-city Los Angeles, health insurance was another factor that determined incomplete immunization [25]. Furthermore, the finding of this study revealed that the availability of health insurance was the determinant of incomplete immunization this was in line with a study conducted in African-American and Latino children in inner-city Los Angeles [25]. Health insurance coverage makes preventive services like vaccinations and screening programs more accessible [26]. The study was not without limitations among those limitations; because the study’s data came from secondary sources, significant variables like environmental and health system-related factors were overlooked. Furthermore, it may be challenging to casually associate partial immunization with the explanatory variable due to the nature of cross-sectional study designs.

## Conclusions

The finding of this study revealed that there was more inter-country variance in the high [27] rate of incomplete vaccination uptake. Completion of vaccination was positively correlated with the mother’s socio-demographic traits, including home delivery, unemployment, lack of education, and non-use of PNC, ANC, and contraception. Living in a rural region was found to be a significant contextual factor related to inadequate immunization in children aged 12 to 23 months in Sub-Saharan Africa (SSA). We strongly advise that the health policymaker try to decrease home delivery and increase maternal health services consumption, such as ANC, PNC, and contraception, to minimize the vaccination incompletion rate. Given that the prevalence of incomplete immunization varies between countries, researchers should carry out more studies to determine the potential cause of variation by including additional explanatory variables, such as factors connected to health care services.

## Data Availability

Data is available online and you can access it from www.measuredhs.com.

## Abbreviations

AOR: Adjusted Odds Ratio
CI: Confidence Interval
DHS: Demographic and Health Survey
ICC: Intra-class Correlation Coefficient
LLR: Log-likelihood Ratio
LMICs: Lower Middle-Income countries
LR: Likelihood Ratio
MOR: Median Odds Ratio
SSA: Sub-Saharan Africa
WHO: World Health Organization
